# Decellularized Scaffolds of Nopal (*Opuntia Ficus-indica*) for Bioengineering in Regenerative Dentistry

**DOI:** 10.3390/jfb14050252

**Published:** 2023-05-01

**Authors:** Ruth Betsabe Zamudio-Ceja, Rene Garcia-Contreras, Patricia Alejandra Chavez-Granados, Benjamin Aranda-Herrera, Hugo Alvarado-Garnica, Carlos A. Jurado, Nicholas G. Fischer

**Affiliations:** 1 Interdisciplinary Research Laboratory, Nanostructures, and Biomaterials Area, National School of Higher Studies (ENES) Leon, National Autonomous University of Mexico (UNAM), Leon 37684, Gto, Mexico; 2 Department of Prosthodontics, The University of Iowa College of Dentistry and Dental Clinics, Iowa City, IA 52242, USA; 3 Minnesota Dental Research Center for Biomaterials and Biomechanics, University of Minnesota, Minneapolis, MN 55455, USA

**Keywords:** cellulose scaffolds, plant-based polymer, three-dimensional cellulose, tissue engineering

## Abstract

*Opuntia Ficus-indica*, or nopal, is traditionally used for its medicinal properties in Mexico. This study aims to decellularize and characterize nopal (*Opuntia Ficus-indica*) scaffolds, assess their degradation and the proliferation of hDPSC, and determine potential pro-inflammatory effects by assessing the expression of cyclooxygenase 1 and 2 (COX-1 and 2). The scaffolds were decellularized using a 0.5% sodium dodecyl sulfate (SDS) solution and confirmed by color, optical microscopy, and SEM. The degradation rates and mechanical properties of the scaffolds were determined by weight and solution absorbances using trypsin and PBS and tensile strength testing. Human dental pulp stem cells (hDPSCs) primary cells were used for scaffold–cell interaction and proliferation assays, as well as an MTT assay to determine proliferation. Proinflammatory protein expression of COX-I and -II was discovered by Western blot assay, and the cultures were induced into a pro-inflammatory state with interleukin 1-β. The nopal scaffolds exhibited a porous structure with an average pore size of 252 ± 77 μm. The decellularized scaffolds showed a 57% reduction in weight loss during hydrolytic degradation and a 70% reduction during enzymatic degradation. There was no difference in tensile strengths between native and decellularized scaffolds (12.5 ± 1 and 11.8 ± 0.5 MPa). Furthermore, hDPSCs showed a significant increase in cell viability of 95% and 106% at 168 h for native and decellularized scaffolds, respectively. The combination of the scaffold and hDPSCs did not cause an increase in the expression of COX-1 and COX-2 proteins. However, when the combination was exposed to IL-1β, there was an increase in the expression of COX-2. This study demonstrates the potential application of nopal scaffolds in tissue engineering and regenerative medicine or dentistry, owing to their structural characteristics, degradation properties, mechanical properties, ability to induce cell proliferation, and lack of enhancement of pro-inflammatory cytokines.

## 1. Introduction

The nopal (*Opuntia Ficus-indica*) is a cactus native to Mexico used since pre-Hispanic times as both food and medicine. Currently, there are 3 million hectares of native nopal and approximately 233,000 hectares cultivated in Mexico [1], making Mexico the world’s leading producer and consumer [2]. It is a shrubby plant with a woody trunk and branches that are formed by cladodes. The nopal tissue has two layers, one consisting of green cells called chlorenchyma, and the second layer is internal and formed by a cylinder of white cells known as parenchyma, in which mucilaginous cells exist, so named for their main function of mucilage storage. The nopal chemical composition on a wet basis is 91% water, 6% carbohydrates, 1.5% cellulose, 1% proteins, and 0.5% fats [1]. Nopal possesses various therapeutic benefits, including lowering postprandial glucose levels in diabetic people due to its high fiber content and enhancing pectin mechanisms that absorb intestinal glucose. It also has antioxidant, analgesic, anti-inflammatory, anti-carcinogenic, anti-viral, and regenerative properties, suggesting that its rich content of carotenoids, flavonoids, phenols, vitamin A, and vitamin E contribute to these effects [1,2].

Natural plant-based scaffold polymers are preferred over synthetic and animal-based alternatives due to their biological property advantages. The role of the scaffold in regenerative engineering is to mimic the complexity required for cell migration, proliferation, and differentiation in tissue regeneration. This is achieved by designing porous scaffolds with specific surface areas, appropriate thickness, good permeability, and mechanical properties [3,4]. The nopal cactus could potentially be utilized in tissue engineering, which commonly involves immersion in decellularizing agents such as sodium dodecyl sulfate (SDS) [5,6] with agitation to create 3D scaffold structures that emulate the functions of original organs or tissues [7]. Natural-cellulose scaffolds, made from sources such as plants, algae, and bacteria, possess unique properties, including biocompatibility, biodegradability, and mechanical properties, that render them suitable for applications in tissue engineering and regenerative medicine [7,8].

In this context, Modulevsky et al. [8] developed 3D scaffolds made of cellulose derived from apples for culturing mammalian cells, demonstrating advantages such as simplification in production and reduced cost compared to other methods. On the other hand, Lee et. al. [9] used decellularized fruits and vegetables such as apples, broccoli, carrots, and peppers to obtain cellulose scaffolds for culturing pluripotent stem cells, demonstrating the ability of the scaffolds to support the in vitro culture of mammalian cells Similarly, Adamski et al. [10] decellularized leaves of *Ficus hispida* using two methods, both of which produced scaffolds with suitable mechanical properties and minimal impact to cellular metabolism.

Moreover, Contessi et al. [11] employed three plant tissues (apple, carrot, and celery) for decellularization, which showed mechanical properties and interconnected pores with a size of 100 to 500 µm, making them favorable for adipose tissue, bone tissue, and tendon regeneration. Recently, Chisci and Fredianelli [12] reported that the enzyme bromelain derived from the stem of the pineapple plant (*Ananas comosus*) was reported to have potential clinical therapeutic applications for preserving alveolar ridge structure. This suggests that bromelain could be a promising candidate for tissue engineering and regenerative dentistry. There are no prior reports in the literature on decellularized nopal cellulose (*Opuntia Ficus-indica*) scaffolds for use in biomedical engineering applications in dentistry or medicine, which presents an opportunity for potential bio-innovation in tissue engineering. Nopal cellulose has shown favorable biocompatibility and biodegradability properties, making it a promising candidate for use in scaffolds for tissue regeneration [13].

The objective of this study was to decellularize and characterize nopal (*Opuntia Ficus-indica*) scaffolds; assess degradation, tensile strength, and the proliferation of human dental pulp stem cells (hDPSCs); and determine the potential pro-inflammatory effects by assessing the expression of cyclooxygenase-1 and -2 (COX-1 and 2). This 3D scaffold system could lead to a potential application in bioengineering.

## 2. Materials and Methods

### 2.1. Nopal Scaffold Synthesis and Decellularization

**Figure 1:**
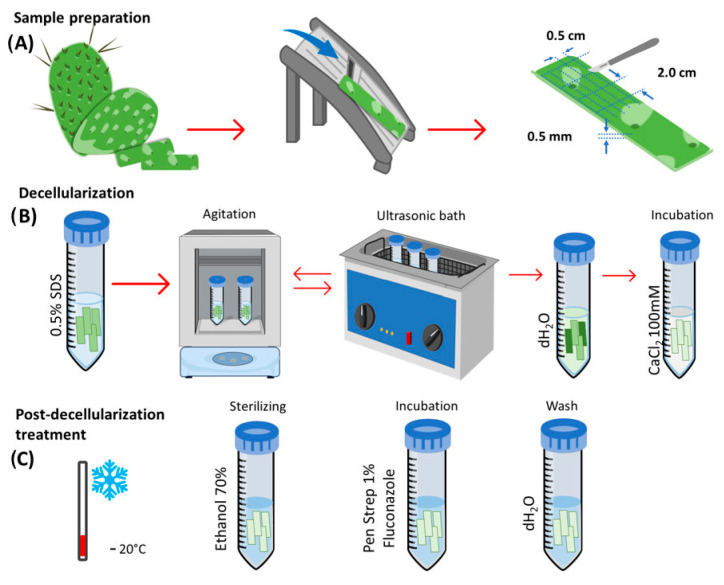
Schematic decellularization of nopal *Opuntia Ficus-indica* scaffold. (**A**) The scaffolds were obtained from nopal grown in a greenhouse located at ENES Leon Unit with coordinates of 12.42385 latitude and −86.881317 longitude. The scaffolds were derived by using the parenchyma (pulp) portion with a mandolin slicer to a uniform thickness of 0.5 cm × 2.0 cm × 0.5 mm. (**B**) Nopal scaffolds were decellularized using 0.5% SDS solution during the 48 h agitation cycle. The resulting structures were washed three times with dH_2_O and incubated in a CaCl_2_ solution. (**C**) Stored at −20 °C, disinfected with a 70% ethanol solution, incubated with PenStrep + 1% fluconazole for 3 h, and washed with sterile dH_2_O (**C**). Abbreviations: SDS = sodium dodecyl sulfate, CaCl_2_ = calcium chloride, PenStrep = penicillin/streptomycin (Sigma-Aldrich).

Scaffolds were obtained from nopal plant tissue (*Opuntia Ficus-indica*) by using the parenchyma (pulp) portion while discarding the chlorenchyma. The nopal was grown in a greenhouse located at the National School of Higher Studies (ENES) Leon Unit with coordinates of 12.42385 latitude and −86.881317 longitude. Initially, parenchyma slices were cut using a mandolin slicer to obtain 0.5 cm × 2.0 cm × 0.5 mm blocks, which were subjected to a decellularization process using a 0.5% SDS solution (sodium dodecyl sulfate, ReagentPlus, Sigma-Aldrich, St. Louis, MO, USA). The SDS solution with the nopal scaffolds was subjected to agitation for 48 h at 180 rpm. After 24 h, agitation was suspended to subject the scaffolds to an ultrasonic bath for 5 min at 40 °C before continuing agitation to complete the 48 h cycle. The resulting structures were then washed three times with dH_2_O and incubated in a CaCl_2_ solution (calcium chloride, Karal, Leon, Guanajuato, Mexico) at a concentration of 100 mM for 24 h at room temperature. The scaffolds were washed three more times with dH_2_O. At this point, both the decellularized scaffolds and native tissue scaffolds corresponding to the study group and control group, respectively, were obtained. Both scaffolds were incubated with penicillin/streptomycin (Sigma-Aldrich) + 1% fluconazole (Laboratorios Senosiain Laboratories, La Piedad, Michoacan, Mexico) for 3 h at 180 rpm. Finally, the samples were disinfected in a 70% ethanol solution for 1 h, washed three times in sterile dH_2_O, and stored at −20 °C until use (Figure 1).

### 2.2. Nopal Scaffold Characterization

The characterization of the scaffolds was carried out by optical microscopy to understand the microstructure and compare the changes that decellularization caused in it. Decellularized scaffolds and native nopal parenchyma tissue were fixed in a 2% glutaraldehyde solution (Karal) and PBS in a 1:1 ratio for 24 h, dehydrated with ethanol gradients at 25, 50, 75, and 100% for 5 min every 2 h in a continuous flow, and stored in a silica mold for 48 h. Subsequently, they were placed on slides to be stained with safranin or coated with a thin carbon layer. The samples were analyzed under an inverted optical microscope (Leica DMIL LED, Deerfield, IL, USA) at 40× and scanning electron microscopy (SEM, JEOL JSM-IT500, Tokyo, Japan) at a magnification of 250× using primary electrons accelerated to 50 kV.

### 2.3. Nopal Scaffolds Degradation

To conduct degradation assays, we used an analytical balance (Denver Instrument, Arvada, CO, USA) to measure the weight of all scaffolds before they underwent the degradation process. This weight was then identified as the initial weight. Afterward, the nopal decellularized and native tissue scaffolds in separate tubes containing a trypsin 0.025% EDTA-2Na in PBS(–) (Sigma-Aldrich) and 1X phosphate buffered saline (PBS) for enzymatic and hydrolytic degradation, respectively. The scaffolds and solutions were placed in Falcon tubes and agitated at 360 rpm at 37 °C for 240 h. Every 24 h, we stopped the agitation to remove the scaffolds from the solutions, record their weight, and measure their absorbance at 350 nm using a UV-Vis spectrophotometer (Multiskan Go, Thermo-Scientific, Helsinki, Finland) to observe proteins in solution.

### 2.4. Nopal Scaffolds tensile Strength

The mechanical properties of native and decellularized nopal scaffolds were assessed using a tensile test, following the ASTM D 882-02: standard test method for tensile properties of thin plastic sheeting. A universal testing machine (Mecmesin, advanced force/torque indicator (AFTI), London, UK) was employed to conduct the tests, with an initial scaffold area of 20 mm × 10 mm positioned between clamps and a cross speed of 1 mm/min applied until failure occurred. Tensile strength was then determined by dividing the force applied to the sample (measured in Newtons) by the sample’s cross-sectional area (in mm) and expressed in megapascals (MPa). The sample size used for this experiment was *n* = 10 per group.

### 2.5. Cell Culture

The hDPSCs were obtained from the cell bank of the Interdisciplinary Research Laboratory, Nanostructures and Biomaterials Area of the ENES Leon, UNAM. The cells were established and characterized as previously reported [14]. Initially, the cells in cryopreservation vials were thawed by placing each vial in the cell incubator for 5 to 10 min. After thawing, the cells were then placed in 10 cm culture plates (Corning Costar^®^, Nagog park acton, MA, USA) with supplemented MEM cell culture medium consisting of 10% fetal bovine serum (FBS), 1% glutamine (Sigma-Aldrich), and 1% antibiotics (PenStrep, Sigma-Aldrich). The cells were then incubated at 37 °C with 5% CO_2_. The culture medium was changed every two days until the cells reached a cellular confluence of over 80%.

### 2.6. Scaffold-Cell Interaction and Proliferation

The hDPSCs cultures (PDL 6) that exhibited confluence higher than 80% were inoculated in 24-well plates (Corning Costar^®^) on scaffolds. The cells were washed with PBS and detached by 0.25% trypsin-0.025% EDTA-2Na in PBS (Sigma-Aldrich) for each experiment. The number of inoculated cells was determined by excluding trypan blue with a hemocytometer under light microscopy. A total of 1 × 10^6^ cells/mL were subcultured on the nopal and native scaffolds. Native nopal tissue was used as the control group. The interaction consisted of 24 h, and the proliferation was incubated from 72 to 168 h at 37 °C with 5% CO_2_ and 95% humidity. The interaction between hDPSCs and nopal scaffolds was examined by looking at the morphological characteristics using both a stereomicroscope (Leica DMIL LED) at 40× and SEM (JEOL JSM-IT500). After the medium was removed, the samples were washed three times with PBS, fixed, stained, dehydrated, and stored, as above mentioned.

The proliferation assay was determined with the MTT method used to determine cell viability. Briefly, the cells were incubated for 7 h in fresh MEM with 10% FBS containing 0.2 mg/mL of Thiazolyl Blue Tetrazolium Bromide (Sigma-Aldrich). After incubation, the formazan produced was dissolved with dimethyl sulfoxide (DMSO, Karal), and the absorbance of the lysate at 570 nm was measured using a microplate spectrophotometer reader (Multiskan go, Thermo-Scientific). Cytotoxicity was assessed according to the ISO 10993-5:2009 standard for in vitro cytotoxicity testing of medical devices.

### 2.7. COX-1 and COX-2 Cell Expression-Scaffold

To determine the protein expression of COX-1 and COX-2, a Western blot assay was performed. A subculture of hDPSCs (1 × 10^6^ cells/mL, 8 PDL) was inoculated with the decellularized nopal scaffolds for 168 h. Subsequently, the cultures were induced into a pro-inflammatory state with 3.12 ng/mL of human interleukin 1-β (IL-1β human recombinant, R&D Systems, Minneapolis, MN, USA) for 24 h. The protein lysis and extraction process were performed with ice-cold 1X RIPA lysis buffer (Biotechnology WVR AMRESCO, Fountain Parkway, Solon, OH, USA) in each scaffold for 15 min at 4 °C. The lysed culture was subjected to sonication for 10 s and centrifugation at 12,000 rpm for 10 min at 4 °C. Protease inhibitor cocktail tablet (Roche Diagnostics, Indianapolis, IN, USA) was used. The protein concentrations in the lysates were determined, and equal amounts of protein for each sample were subjected to 8% SDS-polyacrylamide gel electrophoresis and transferred to a polyvinylidene membrane (PVDF, immobilon^®^-P Transfer Membranes, Sigma-Aldrich). The membrane was blocked with a 3% skim milk solution for 2 h while stirring at 25 °C. The membrane was incubated with monoclonal antibody anti-COX-1 or anti-COX-2 (Santa Cruz Biotechnology, Dalla, TX, USA) or β-actin (Sigma-Aldrich), diluted 1:1000 in 3% blocking solution for 1–2 h, followed by horseradish peroxidase-coupled anti-mouse IgG polyclonal antibody (Sigma-Aldrich) diluted 1:1000 in 3% blocking solution for 1 h. The visualization of the complexes formed was performed by chemiluminescence using Clarity max Western ECL substrate (Bio-Rad, Hercules, CA, USA), and the results were imaged using the C-Digit Blot scanner (Li-Cor, Lincoln, NE, USA) and analyzed in Image Studio Version 4.0 software.

### 2.8. Statistical Analysis

The data represent mean ± standard deviation and were analyzed with the Shapiro–Wilks normality test, *t*-student, and ANOVA post-hoc Tukey test. The significance was considered at *p* < 0.05 with a confidence interval of 95%.

## 3. Results

### 3.1. Nopal Scaffold Characterization

**Figure 2:**
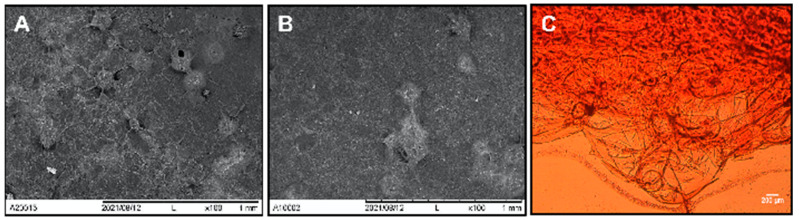
Nopal (*Opuntia Ficus-indica*) decellularized scaffolds and hDPSCs–scaffold interaction. The nopal scaffold decellularization process was achieved using a 0.5% SDS. Native nopal parenchyma tissue (**A**) and decellularized scaffolds (**B**,**C**) were fixed in a 2% glutaraldehyde solution for 2 h and dehydrated with ethanol gradients at 25, 50, 75, and 100% for 5 min each. Subsequently, the sample was placed on slides, stained with safranin, and observed under an inverted optical microscope at 40×. For SEM, the samples were coated with a thin carbon layer and imaged at a magnification of 250× using primary electrons accelerated to 50 kV. Abbreviations: SDS = sodium dodecyl sulfate, SEM = scanning electron microscope. Scale bars are indicated in each panel.

The decellularization method using a 0.5% SDS solution proved to be effective in isolating the extracellular matrix of the nopal after 48 h, ensuring complete removal of the cells. This was immediately confirmed as the obtained samples appeared colorless and translucent. Decellularization was also verified through optical microscopy and SEM, where micrographs of decellularized samples were compared with those of native tissue without decellularization. Optical microscopy and SEM showed images that corresponded to a porous structure of 252 ± 77 μm (*n* = 9, 150 to 400 μm) with interconnected circular shapes, positive for cellulose (Figure 2A–C). The micrograph of the native tissue showed spherical, clustered structures where the plant tissue cells were suggested to be located. In the decellularized tissue sample, this bulging of circular structures was not observed. Instead, a porous structure was seen, indicating the absence of plant cells, thus, achieving decellularization and the isolation of the cellulose nopal extracellular matrix.

### 3.2. Nopal Scaffolds Degradation

**Figure 3:**
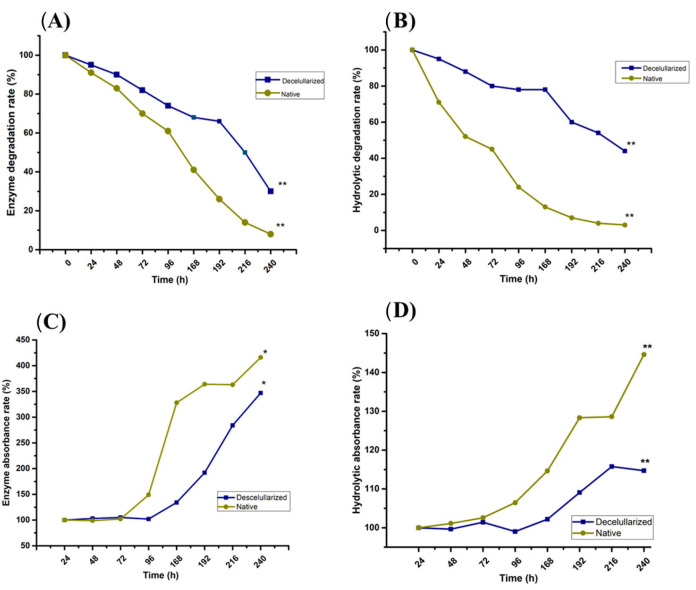
Nopal decellularized scaffold or native tissue enzymatic and hydrolytic degradation. The scaffold’s initial and final weights were recorded from 0 to 240 h after being incubated under agitation for 360 rpm at 37 °C in trypsin-0.025% EDTA-2Na in PBS (**A**) and PBS (**B**). The weight was determined every 24 h and the absorbance of the solution was measured at 350 nm using a UV-Vis spectrophotometer (**C**, trypsin and **D**, PBS). Nopal decellularized scaffolds correspond to a 57% weight loss in hydrolytic degradation and 70% in enzymatic degradation. Each value represents the mean ± SD of triplicate assays (*n* = 9), * *p* < 0.05, ** *p* < 0.001 Student’s *t*-test. Abbreviations: EDTA = ethylenediaminetetraacetic acid, PBS = phosphate buffer saline solution, SD = standard deviation.

The degradation of the decellularized scaffolds or native tissue was evaluated by comparing the initial and final weights and measuring the absorbance of the solutions. The initial weight was identified as the first measurement at 0 h of the degradation process, and the final weight was the measurement taken after 240 h of agitation in trypsin and PBS solutions. During the process, it was evident that the scaffold structure’s dimensions decreased, and the weight measurement demonstrated a gradual decrease over time, registering a 57% weight loss for the decellularized scaffolds in hydrolytic degradation and a 70% weight loss in enzymatic degradation, with a statistically significant difference (*p* < 0.01) compared to the degradation of native tissue scaffolds (*n* = 9). The degradation was evaluated up to 240 h because it was impossible to manipulate the scaffolds after this time due to their fragmentation and degradation (Figure 3A,B). In the measurement of absorbance using UV-Vis spectrophotometry at 350 nm, an absorbance increase was observed after 96 h in native and decellularized scaffolds (*p* < 0.01, *n* = 9). The native tissue exhibits more solution absorbances correlated to the degradation process in both hydrolytic and enzymatic solution (Figure 3C,D).

### 3.3. Scaffold Tensile Strength

The assessment of mechanical properties of tensile strength indicated that both native and decellularized nopal scaffolds exhibited values of 12.5 ± 1 MPa and 11.8 ± 0.5 MPa, respectively. Notably, statistical analysis revealed no significant difference between the two groups (*n* = 10, *p* > 0.05), suggesting that both types of scaffolds possess comparable tensile strength (Table 1).

### 3.4. Cell-Scaffold Interaction and Proliferation

**Figure 4:**
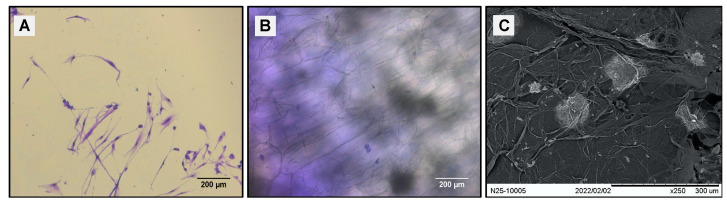
Initial hDPSCs–nopal scaffold interactions. In the case of the hDPSCs–nopal scaffold interaction, near confluent hDPSC cells (80% confluence, 6 PDL) were incubated as control on a plastic dish (**A**) and then on the nopal decellularized scaffold for 24 h (**B**,**C**). After incubation, the medium was removed and treated as above mentioned for an inverted optical microscope and SEM. hDPSCs = human dental pulp stem cells, SDS = sodium dodecyl sulfate, SEM = scanning electron microscope. Scale bars are indicated in each panel.

**Figure 5:**
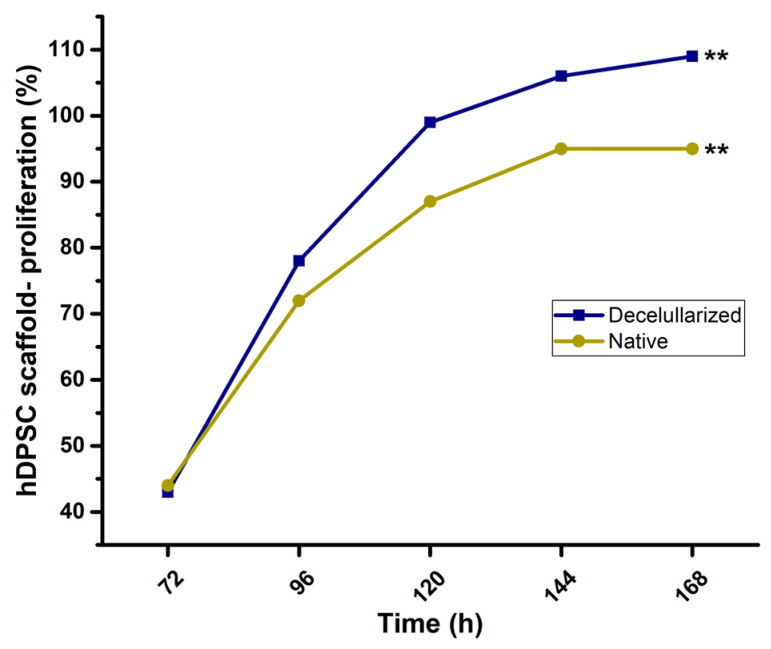
hDPSCs–nopal scaffold proliferation. hDPSCs at 1 × 10^6^ cells/mL (6 PDL) were inoculated over the nopal and native scaffolds from 72 to 168 h at 37 °C with 5% CO_2_ and 95% humidity. The hDPSCs proliferation was determined by the MTT method (0.2 mg/mL). The nopal decellularized scaffold showed a major number of proliferated hDPSC at 168 h when compared to native tissue. Each value represents mean ± SD of triplicate assays (*n* = 9), ** *p* < 0.001 Student’s *t*-test. Abbreviations: hDPSCs = human dental pulp stem cells, PDL = population doubling level, MTT = 3-[4,5-dimethylthiazol-2yl]-2,5-diphenyltetrazolium bromide, SD = standard deviation.

Images of 2D cultures on culture plates alone as a control showed cells exhibiting a fusiform elongated and flattened morphology (Figure 4A). On the other hand, the hDPSCs–nopal decellularized scaffold interaction showed cells with a spherical shape on the scaffold (Figure 4B). Similarly, samples were prepared for characterization by SEM, which confirmed the results obtained with optical microscopy, showing cells with a circular and spherical morphology on the scaffolds (Figure 4C). The decellularized nopal tissue scaffolds present an optimal microstructure for hDPSCs to adhere and proliferate. hDPSCs exhibited significantly exponential cell viability (*n* = 9, *p* < 0.01) at 168 h compared to native tissue (Figure 5) with 95% and 106% viable cell number for native and decellularized scaffolds.

### 3.5. COX-1 and COX-2 Cell Expression-Scaffold

**Figure 6:**
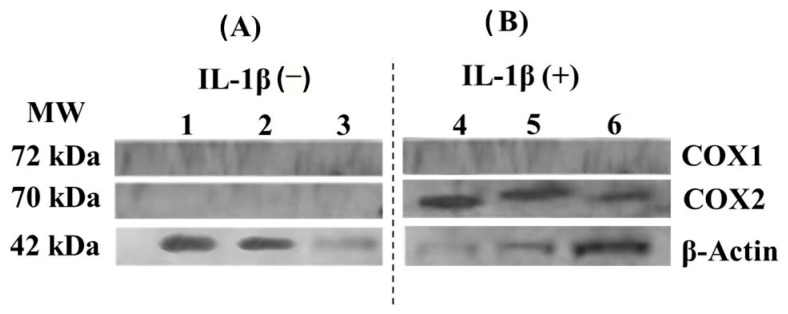
Expression of pro-inflammatory proteins COX 1 and COX 2 of hDPSCs–decellularized nopal scaffolds interaction. hDPSCs at 1 × 10^6^ cells/mL (8 PDL) were inoculated over the decellularized nopal for 168 h. Subsequently, the cultures were induced into a pro-inflammatory state (**B**, IL-1β = 3.12 ng/mL) or not (**A**) for 24 h. The protein lysis and extraction process were performed with RIPA lysis buffer and the Western blot protocol was performed. The nopal scaffold did not cause any noticeable increase in COX-1 and COX-2 protein expression (**A**). However, when combined with IL-1β, the pro-inflammatory state enhanced the expression of COX-2 (**B**), and β-actin was used as an internal positive control. Abbreviations: MW = molecular weight marker, COX = cyclooxygenase, hDPSCs = human dental pulp stem cells, PDL = population doubling level, IL-1β = human interleukin 1-β.

The interaction of decellularized nopal scaffold and hDPSCs did not cause any noticeable increase in COX-1 and COX-2 protein expression (Figure 6A). However, when combined with IL-1β, a known pro-inflammatory stimulator, the expression of COX-2 was increased, as shown in Figure 6; β-actin was used as an internal control. Based on these findings, it can be concluded that the scaffold by itself does not induce the pro-inflammatory expression of COX-1 or COX-2 but permits physiologic cell function under IL-1β stimulation.

## 4. Discussion

### 4.1. Scaffold Decellularization

The method of decellularizing plant tissue described in this study, using a 0.5% SDS solution and agitation, represents a safe, simple, and economical procedure that requires minimal resources. As a result, it is environmentally friendly and helps preserve the extracellular matrix of plant tissue [5,15]. This makes it a favorable method as it allows for cellular adhesion and proliferation on scaffolds and nutrient transfer through the nopal tissue and can, therefore, be used in tissue engineering. Contessi et al. [11] conducted the previous research demonstrating the potential of decellularized apple, carrot, and celery tissues for regenerating adipose, bone, and tendon tissue. This was attributed to their mechanical properties and interconnected pores, ranging from 100–500 µm, which were found to be optimal for cellular migration, proliferation, and differentiation. Our present study similarly found that decellularized nopal tissue scaffolds exhibited a porous structure with interconnected circular shapes with a diameter of 252 ± 77 μm, suggesting that decellularized nopal tissue could also have potential for tissue regeneration.

### 4.2. Nopal Scaffolds Degradation

Cellulose possesses the ability to persist at the implantation site for extended periods due to its resistance to enzymatic degradation by mammalian cells [16]. However, this can be altered by adjusting the degradation rate through hydrolysis pretreatment and by administering cellulases or collagenase along with the scaffolds. Regarding the degradation process, a weight loss of 70% was observed in the decellularized scaffolds, which coincides with the report by Modulesvky et al. [17] for subcutaneous implantation of decellularized plant tissue scaffolds, which can demonstrate their possible degradability. In this study, the scaffolds showed significant degradation under hydrolytic and enzymatic conditions with PBS and trypsin over a period of 240 h, as evidenced by changes in weight loss and solution absorbance, in contrast to native tissue. These findings suggest that decellularized plant cellulose-based scaffolds hold promise as an alternative to traditional animal-derived scaffolds for tissue engineering purposes. However, further research is required to fully evaluate their long-term biocompatibility and degradation.

### 4.3. Nopal Scaffold Tensile Strength

Previous studies have evaluated the mechanical properties of natural scaffolds based on aloe vera and cellulose alone or in combination with other polymers for bone tissue engineering. Khoshgozaran-Abras et al. [18] and Saibuatongbased and Phisalaphong [19] have reported these scaffolds can effectively improve the mechanical properties, based on standard ASTM D 882-02. For instance, aloe vera combined with hydroxyapatite was shown to increase the reported tensile strength from 4.80 MPa to 10.89 MPa. Additionally, Bahaaraty et al. [20] evaluated the elastic modulus in nanofiber scaffolds based on copolymer poly(l-lactic acid)-co-poly (ε-caprolactone) (PLACL), silk fibroin (SF), and aloe vera. The results showed elastic modulus values of 14.1 ± 0.7, 9.96 ± 2.5, and 7.0 ± 0.9 MPa, respectively. In comparison, the nopal decellularized scaffolds in the tensile strength test exhibited higher values than the previously reported results, indicating that nopal tissue enhances the mechanical properties and may be comparable with synthetic scaffolds. However, when Bielli et al. [21] evaluated the tensile strength of collagen fiber scaffolds from bovine pericardium (biomeshes), the values ranged from 21.44 to 50.91 MPa, suggesting that the biomeshes possess higher tensile strength compared to our results.

### 4.4. Cell-Scaffold Interaction and Proliferation

Proliferation assays of hDPSCs carried out in this study demonstrated an exponential-constant increase in viability with a statistically significant difference in proliferation between hDPSCs on decellularized scaffolds and native tissue. This was also observed in previous reports where decellularized plant tissue scaffolds were used for the culture of different mammalian cells [6,7,11,17,22]. With these results, it can be said that there was in vitro biocompatibility of the cells with the decellularized nopal scaffold, as it was shown that they were able to adhere, invade, and proliferate within the cellulose scaffolds, indicating that this tissue had high viability even after 168 h. The morphological characteristics of hDPSCs on decellularized nopal scaffolds observed in the micrographs show oval, circular, and spherical-shaped cells, unlike 2D cultures that are flattened and elongated. This is the trait that makes them different from 2D cultures, and these results have similarities with what other authors have reported [23,24]. The importance of morphology in 3D cultures is that it allows conditions to be imitated and behaviors to be replicated in vivo. Three-dimensional culture systems aim to replicate the microstructures of organs, using the extracellular matrix (ECM) as scaffolding. This approach was used in organoids of various organs [25]. Future experiments should prioritize the incorporation of extracellular matrix components to promote the development of bone, adipose, and cartilage tissue initially before moving on to creating multipotential scaffolds for other tissues in the field of regenerative engineering.

### 4.5. COX-1 and COX-2 Cell Expression-Scaffold

Finally, in evaluating the potential inflammatory effect of decellularized nopal scaffolds, the results obtained were that the interaction of hDPSCs with decellularized nopal scaffolds did not provoke an inflammatory state in the cultures. These were favorable results as it has potential clinical application as there is no foreign body rejection. This can be compared to what Yi et al. 2020 reported, where they conducted a Western blot to determine the anti-inflammatory effect by identifying the expression of cytokines such as tumor necrosis factor α in mesenchymal cell culture models [26]. Biocompatibility is an essential aspect to consider when using biomaterials, as it refers to their ability to interact with living tissues without causing adverse effects such as inflammation or toxicity. Previous studies have evaluated the biocompatibility of various biomaterials [27] for different biomedical applications, including hip prostheses, mechanical properties [28], and clinical or simulated interventions [29]. In this study, we demonstrate that nopal decellularized scaffolds exhibit neither cytotoxicity nor inflammation in vitro, suggesting their potential use in biomedical applications. Therefore, it is crucial to carefully consider the nature, mechanical properties, and biocompatibility of biomaterials when selecting suitable candidates for specific biomedical applications and to evaluate their safety and efficacy through clinical or simulated assessments.

### 4.6. Limitations of the Study

One limitation of this study could be related to the geographical location and cultural variations of nopal (*Opuntia Ficus-indica*), which may impact the reproducibility and scalability of scaffold production for industrial applications in tissue engineering. Standardizing conditions could help mitigate this limitation. Another limitation is that despite the incorporation of antifungal agents in the culture medium, slight fungal infections were observed after 15 days of culture. This highlights the need for further research to optimize the antifungal properties of the scaffolds to ensure their long-term stability in tissue engineering applications.

## 5. Conclusions

These findings indicate that the nopal (*Opuntia Ficus-indica*) scaffolds were effectively decellularized using 0.5% dodecyl sodium sulfate, while retaining the extracellular matrix of cellulose. The scaffolds were able to degrade by 50–70% at 240 h, and when human dental pulp stem cells (hDPSCs) were cultured on the scaffolds, they exhibited significant proliferation without affecting cell interactions or the expression of the pro-inflammatory proteins COX-1 and -2. In summary, the use of decellularized nopal scaffolds holds great promise for regenerative dentistry and medicine. Further experiments will focus on evaluating the interaction between the cell–scaffold interaction by investigating focal adhesion kinase signaling, integrins, and other extracellular matrix proteins to better understand cell adhesion, migration, and differentiation with different types of cells. Other cell types, such as bone marrow stem cells, should also be explored. In addition, efforts will be made to increase the mechanical properties of the scaffold, and micro-CT evaluation will be conducted to understand the ultrastructural topography and predict the biological mechanisms involved. Potential further studies can utilize computational simulation [30], in silico approaches [31], or a contact pressure 3D model [32] to study the performance of nopal scaffolds effectively and accessibly and predict their results, as compared to traditional experimental testing, in vitro and in vivo or clinical study.

## Figures and Tables

**Table 1:** Comparison of tensile strength of native and decellularized nopal (*Opuntia Ficus-indica)* scaffolds (*n* = 10 per group).

Scaffold	Tensile Strength (MPa)	*t*-Student Test
Native nopal scaffold	12.5 ± 1	*p* = 0.0748
Decellularized nopal scaffold	11.8 ± 0.5	
MPa = Megapascals		

## Data Availability

The data presented in this study are available on request from the corresponding author.
